# Web messaging among young people in online services: A descriptive mixed-methods study

**DOI:** 10.1177/20552076221092534

**Published:** 2022-04-11

**Authors:** Kiki Metsäranta, Minna Anttila, Tatjana Pajamäki, Heidi Holappa, Maritta Välimäki

**Affiliations:** 1 Department of Nursing Science, Faculty of Medicine, 8058 University of Turku, Turku, Finland; 2 Helsinki University Hospital and University of Helsinki, Helsinki, Finland; 3 Child and Youth Phone and Online Services, Mannerheim League for Child Welfare (MLL), Helsinki, Finland; 4 Xiangya School of Nursing, 12570 Central South University, Changsha, Hunan, China

**Keywords:** web messaging, young people, internet, online, asynchronous, two-way communication, counselling

## Abstract

**Objective:**

To describe web messaging patterns and the content of web messages among young people in a Finnish national online service.

**Methods:**

A descriptive mixed-method was used. The data consisted of text-based web messaging communication between young people and a counsellor in a nationwide online service between 1 January and 31 December 2018. Web messaging patterns were analysed using descriptive statistics. The content of the messages was analysed with thematic analysis and qualitative results were presented. In addition, the factors associated with messaging patterns and content were analysed.

**Results:**

A total of 1941 messages were sent by 1354 young people. Most of them were between 12 and 17 years old and females. Less than one-fifth of young people had multiple two-way discussions with counsellor. The total period of two-way discussions and the number of words in each message varied widely. The number of words was lower in messages sent by males. The content of the messages was divided into three main themes: interpersonal relationships and environment (Social relationships), oneself (Construction of self), and health-related problems and support received from professionals (Health and wellbeing). The young people’s messages mostly contained topics related to the main theme of ‘Social environment’.

**Conclusion:**

Most young people sent one message only. Messages ranged from simple, single messages to complex texts describing the daily life of young people. Girls were more active in messaging, and they wrote longer texts.

## Introduction

The mental health of young people is a global concern.^1^ Typically, mental health problems develop in a person before they reach the age of 14 years,^2^ and their estimated prevalence is around 10–20%.^1^ It has been estimated that the global cost of mental health problems in the year 2010 was US$2.5 trillion (5% of the global gross domestic product),^3^ and the costs are predicted to increase six-fold over the coming 30 years.^4^ Therefore, early support for young people is needed to reduce the likelihood of them developing more severe problems in the future.^5,6^

A number of online services have already been developed for young people through which they can share information and receive support for mental health challenges^7^ such as depression, anxiety, self-harm behaviour and suicidal ideation.^6–8^ Examples of online services include online chat,^7,9–11^ text messaging,^12,13^ asynchronous text-based services,^14^ and websites through which users can post questions and receive answers publicly.^6,8,15^ In these text-based online services, young people discuss their interpersonal relationships, sexuality and sex-related issues,^7,8^ physical maturation, self-esteem^8^ and suicidal ideation.^6^ Text-based online services can ease the burden that young people feel^6,11^ and increase their feelings of hope.^10^ In general, young people are familiar with seeking help online for their mental health issues.^16^ They also appreciate receiving written responses from online services.^6^

There is some discussion in the literature on how revealing patterns of the use of online services, such as the frequency and length of messages, could help in understanding the significance of web messaging in young people’s lives.^12^ One study of post-traumatic stress disorder found that the more patients sent messages, the more likely they were to commit to treatment.^17^ Another study on an online counselling service for young people showed that the messages related to mental health or suicide were longer than the messages containing other topics.^8^ Although it has been found that boys tend to find it easier to seek help from online services than to seek face-to-face treatment,^18,19^ girls with depressive symptoms are generally active online services users,^5^ and they are more familiar with writing into text-based online services.^5,6,11^ However, the importance of the length of the content of a text message or chat between a young person and a counsellor is somewhat unclear.^9,13^ One study examining a counselling service showed that the number of online chats a young person participated in did not significantly affect their psychological distress or life satisfaction, although those who used online chat one to five times during a six-week period reported higher levels of hope than those with no chat counselling.^10^

The topics young people address in online messages vary from interpersonal relationships to serious mental health concerns.^6,7^ With anonymous online services, the topics seem to be sensitive, emotional and challenging.^7,8,11,15^ Topics posted in online services can include relationships,^6–8,11,15^ home or school situations, mental or physical health,^6–8^ body and appearance,^8^ sex, substance use^6,8,11,15^ or suicidal thoughts.^6^ Analysis of the content of the website messages has shown that, typically, each message includes more than just one topic.^6,8^ Multifaceted content of young people’s web messages could also represent their complex life situations.^6^ Therefore, a combination of quantitative and qualitative studies might offer a more complete understanding of young people’s lives.^20^

Despite a wide variety of existing online services, little is known about the pattern of online website messages used among young people, the content of their messages, or the factors associated with the use and content of their online web messaging. Filling this knowledge gap would be important; for example, the number of messages can indicate a young person’s desire for help and their need for clarification of their situation,^17^ while the length of messages can indicate if a young person is having mental health problems or suicidal thoughts.^8^ Therefore, the aim of this study is to describe the frequency and patters of web messaging among young people. Qualitative methods were used to identify the topics of young people’s web messages. In addition, the factors associated with messaging patterns and topics of web messages were analysed with quantitative methods. The data were collected by a Finnish online service of the Mannerheim League for Child Welfare (MLL), the ‘Child and Youth Helpline’. As far as we are aware, this is the first nationwide study to analyse messaging patterns on a publicly available online web messaging service targeted at young people. In this study, web messaging is defined as anonymous, continuous, free-form, and secure two-way written messaging between a young person and a trained adult volunteer.^21^

## Methods

### Design

A population-based descriptive study design was used. This was suitable for our purpose as our analysis included all the web messages sent by young people.^22^ In the data analysis, a descriptive mixed-methods approach was used. Results from the quantitative and qualitative data were used to achieve a more complete understanding than could be provided by the quantitative or the qualitative results alone.^20^ The quantitative data describe the pattern of how web messaging was used (the number of messages, length of messaging periods in days, and the number of words in each message), while the qualitative data describe the content of the written texts.^23^ Further, the qualitative data have been thematised and further quantified to describe the frequency of each theme in the data.^20,24,25^ In addition, factors associated with messaging patterns and content have been analysed.

### Setting

The data were collected from MLL’s online service targeting young people. Through the ‘Youth Online Services website, young people have access to the ‘Child and Youth Helpline’, which offers online counselling. The helpline is an anonymous online contact channel that uses web messaging (asynchronous) and web chat (synchronous) and provides supportive counselling in response to young people’s needs. Volunteer counsellors are adults trained in their tasks by professional instructors at MLL.^26^ The web messaging service, established in 2002, is available 24 h a day, every day. Since 2018, MLL has maintained the web messaging service, which offers multiple two-way communication. The service allows the development of a long-term relationship between a young person and a counsellor in text format. To ensure confidentiality and secured communication, it uses restricted access and password-protected pseudonyms. Each young person creates their own confidential account. Young people can write their messages at any time on any topic. A counsellor responds to the message in the order of message arrival within two weeks via an online mailbox. In 2018, the average response time was 4.6 days (SD 3.25). The messages sent between a young person and a counsellor form a single thread. The account on the website is valid for 30 days after the young person has read the counsellor’s response. If the young person sends a new message during these 30 days, the account will remain valid for another 30 days from when the last message was sent.

### Eligibility criteria

The data comprised written web messages in the ‘Child and Youth Helpline’ sent by young people to MLL between 1 January and 31 December 2018. We included messages that were written in Finnish by a young person themselves. Messages were excluded if they were written by a parent, if the content was a question related to MLL services, if the message was empty, duplicated or used for testing purposes only, or if the sender had prohibited in writing the use of the message for research purposes. Counsellors’ responses were not included in this study.

### Data collection

The data were collected in a single phase.^27^ Automatically collected information about each message was extracted from the data: age of the message sender (≤11, 12–14, 15–17, ≥18), gender (girl, boy, other) and residential area (urban, rural). We also collected the season (month), weekday, and time (hour) that the messages were sent. The use of web messaging was extracted in the data as follows: the number of messages sent by one person, the length of the messaging period in days (starting and ending day, month), and the number of words in each message.

**Figure 1.**
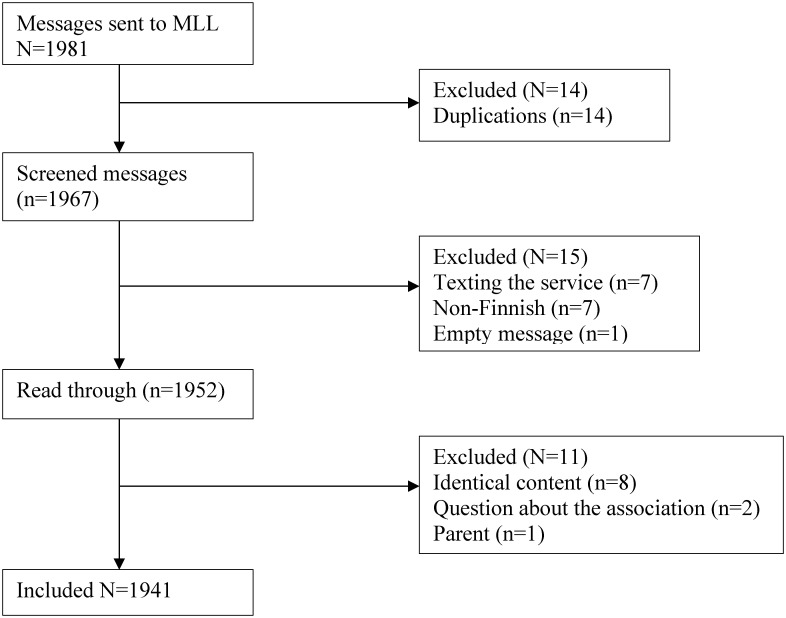
Flow chart of messages.

The data were transferred from MLL to one of the researchers in Excel format. All messages were screened according to the eligibility criteria. Out of 1371 participants who sent 1981 messages, 22 (1.6%) participants and 22 (1.1%) messages were excluded: messages sent were duplications, were only sent to test the service (*n* = 4), message was not in Finnish (*n* = 1), one message contained no content (*n* = 1), participants were asking for information about the association (*n* = 2), and message was sent by a parent (*n* = 1). Out of the original data, a total of 1941 messages (98%) sent by 1354 (99%) young people were included in the analysis. The data included 454,837 words. A detailed flow chart of the messages is presented in Figure 1.

### Data analysis

The quantitative data were transferred into Excel format by the service provider. To count the words used, writings were converted into Word document. The data were managed and analysed in Finnish.

Descriptive quantitative analysis (frequencies, percentages, means with SD for normally distributed variables, medians with ranges for variables of skewed distribution) was used^22^ to describe the background information of the participants (age, gender, residential area) and how the young people used web messaging (number of messages, length of messaging, number of words). To understand the patterns of the young people’s messaging, the times that the messages were sent (season, day, time of day) were analysed. To indicate the activity of the web messaging, the frequency of messages sent was classified into one of two groups: one message or at least two messages. Seasonal variation was analysed in three-month periods: winter (January–March), spring (April–June), summer (July–September) and autumn (October–December).^28^ Variation in days of the week that messages were sent was also analysed (Sunday, Monday, Tuesday, Wednesday, Thursday, Friday, Saturday). To look for a pattern regarding the time of day messages were sent, 24 h was divided into three time frames: night time, when most Finnish youth are in bed (11 PM–7 AM)^29^; day time, when young people are usually active at school (7 AM–3 PM),^30^ and evening time, which included the time after school, when young people might be preparing for the next day (3 PM–11 PM). Young people are generally most active online in the evenings.^31^

Normality of the data was tested using the Kolmogorov-Smirnov test. Outliers in the data were checked but not identified. The variables describing messaging patterns were not normally distributed, and therefore the Mann-Whitney *U* test or Kruskal-Wallis test was used (followed by post hoc comparisons using Bonferroni corrections). A *p* value of 0.05 or less was used to indicate a statistically significant difference in the data. Effect sizes were used as supplement tests of significance.^32^ Effect sizes were estimated using epsilon (ε^2^) and eta squared (η^2^) with benchmarks for small, medium, and large effects being <0.01, <0.06 and >0.14, respectively.^33^ Factors associated with these messaging patterns (season, weekday and time of day) and content were analysed with Pearson’s Chi-square and Fisher’s exact tests (followed by post hoc comparisons using Bonferroni corrections). A *p* value of 0.05 or less was used to indicate a statistically significant difference in the data. Effect sizes were estimated using Cramer’s V (0.1–0.3 small, 0.3–0.5 medium, 0.5 large)^34^ or calculated odds ratio (OR) (small 1.5, medium 2, large 3).^32^ The analyses were conducted using SPSS (version 25.0; IBM Corp).

For the qualitative data, an iterative step-by-step thematic analysis was carried out^23,35^ by two female researchers (KM, MA) who were used to working together. KM is a psychiatric nurse with a master’s degree and long-term experience in psychiatric nursing with young people, and MA is a healthcare professional with a PhD and long-term research experience in nursing science and with conducting qualitative analysis. The researchers had no previous relationships with the participants or preconceptions of the data.

Iterative analysis suited our purpose of processing a large amount of text data. The themes were inductively defined from the raw data without predetermined classifications.^23^ The data were managed in Word format and NVivo software (version 12 for Windows).

The iterative analysis was conducted in seven steps as follows. First, a researcher (KM) read all messages (Word format 1051 pages in Times New Roman 12-point font and 1.5 line spacing) to become familiar with the information and to develop a general understanding of the content of the data. Second, open coding was used, and initial codes were generated. Two researchers (KM, MA) individually coded the first ten messages,^36,37^ which were used to create an analytical frame (e.g. codebook)^37–40^ and to establish a reliable process. The coding results were compared between the researchers to identify differences and similarities in the coding system.^41^ All messages were then read again to develop an accurate coding system. The coding was saturated when no new codes were identified.^37,38^ Any discrepancies were negotiated and resolved with the third author (MV).^36,37^

Third, the codes were inductively grouped into categories according to their similarities. We used the Framework Method, which is a usable method for categorising and organising data, and for developing an analytical framework.^38^ Thus, the data (messages 1–30) comprised 42 codes within 11 categories and their definitions, which formed the codebook.^37,39,40^

Fourth, the reliability of coding with a codebook was assessed in a pilot test using the intercoder reliability (ICR) of the 30 messages (31–60)^42^ to receive information about the consistency between the authors.^39,40^ The agreement between the researchers was calculated for all 11 categories^39^ using Cohen’s kappa.^40,43^ The similarity between the researchers’ coding based on specific words or sentences^39^ was assessed using the simple proportion agreement (e.g. ratio of agreements to disagreements).^36,39^ The classification of quotations led to a Cohen’s kappa coefficient of 0.80 on a scale of out of −1.00 to 1.00,^43^ while the simple proportion agreement was 0.73. Based on these results, we assumed that the ICR was acceptable.^36,39^

Fifth, the rest of the messages were coded by KM according to the codebook.^36–38^ The data were transferred into a format where one message formed a single file in NVivo 12 Plus QSR International. Only one new code (sexuality) was added to the codebook.^37,38^

Sixth, categories were organised into seven subthemes (Interpersonal relationships; Living environment; Past self, Present self and Future self; Health-related problems; Experiences and perceptions of seeking and receiving help from professionals) and three main themes (Social environment; Construction of self; Health and well-being) to depict the data. Researchers again categorised all phrases or paragraphs that corresponded to each content area of the codebook and formed themes. The extent to which the themes contributed to the content of the data was examined. The data were interpreted into a larger context using a deductive theoretical framework to help further explain^38^ the young people’s perceptions of themselves.^44,45^ After the thematic analysis, the occurrence of each theme in the data was calculated,^24,25^ and factors associated with background information and the number of themes identified in the data were analysed.

Finally, the analysis report was written, which offered answers for the study objective. Robustness of the results was ensured by using quotations from the original data to illustrate specific themes. The number of each quotation represents an original ID code of a specific young person. A native English speaker checked all quotations to ensure correct interpretations of the data. Further, after thorough reflection and discussions between all co-authors, the study results and quotations were presented and shared with professional instructors at MLL to ensure that the results represented the actual situation.^41^

### Ethics

Ethical approval was granted by the HUS Ethics Committee (Diary code 1759/2019). A research permit was obtained from MLL (Date 20.06.2019). The ethical principles of privacy and data protection were respected so that it would not be possible to identify individual participants^46^ as the data to which we had access were in an anonymous format. Information on the login page of the ‘Child and Youth Helpline’ stated that texts written by young people could be used for research purposes.

### Validity and reliability

To increase the validity of the analysis of large and rich text data, we used the Framework Method to develop a codebook.^38^ A codebook as a data categorisation tool has been reported to be a usable and valid method for developing a practical and accurate tool for guiding the analysis of a large amount of qualitative data, operationalising codes, and improving the consistency of coding.^37^ In our case, the codebook made it possible to understand the content of the messages. To increase the validity of the codebook development, we used ICR to assess the reliability of the codebook^36,42^ and to increase the post hoc evaluation of our study.^47^

We also used a theoretical framework, *self-concept*, to help further explain the main theme ‘Construction of self’.^38^ We verified that self-concept was appropriate to use as a theoretical framework^47^ to divide young people’s perceptions of themselves into past self, present self and future self. Self-concept is used here to encompass how a person’s self-perceptions are formed through experiences in and interpretations of their environment. The term also considers the perception of oneself that influences the ways one acts, which, in turn, influences the ways that one perceives oneself.^45^ It is based on past behaviour, and it can predict future behaviour. It can be descriptive and evaluative.^44^ Further, the authors’ conclusions made in all phases of the study involved moving back and forth between the original data and decisions made. Results were validated with members of MLL, and the results corresponded to their experience with message content.^41^

## Results

### Characteristics of the participants

Altogether, 1941 messages were sent by 1354 participants. Out of 1354 participants, 47.4% (*n* = 642/1354) were in the age group of 15–17-year-olds. Females made up 87.0% (*n* = 1180/1354), and 70.7% (*n* = 959/1354) lived in an urban area (Table 1).

**Table 1.** Characteristics of the young people (*N* = 1354) who used web messaging.

Characteristics	*n*	%
Age		
≤11	64	4.7
12–14	440	32.5
15–17	642	47.4
≥18	208	15.4
Gender		
Female	1178	87.0
Male	119	8.8
Other	57	4.2
Residential		
Urban	957	70.7
Rural	397	29.3

### Use of web messaging

A total of 1941 messages were sent by young people. The time period of sending at least two messages (i.e. time between first and last message sent by a young person) ranged from 1 to 294 days (Median 15). The number of words in each message varied from 2 to 4097 words (Median 168) (Table 2). Out of all the young people in the data, 82.3% (*n* = 1115/1354) sent only one message, while 17.7% (*n* = 239/1354) sent at least two messages.

**Table 2.** Description of the number of messages sent, the length of messaging period and the number of words in each message.

Variables	*N*	Min	Mean	SD	Max	Median	Mode
Number of messages	1941	1	1.43	1.85	31	1	1
Length of messaging period	239	1	44.14	64.51	294	15	5
Number of words	454,837	2	234.33	242.97	4097	168	54

There were some variations in seasons and the number of messages sent. The fewest messages were sent in summer (July–September): 18.0% (349/1941). In terms of days of the week, fewer messages were sent on Sundays (279/1941, 14.4%), although these numbers did not vary greatly. Regarding time of day, over half of the messages were sent during the evening (*n* = 1243/1941; 64.0%), and the fewest were sent at night (*n* = 107/1941; 5.5%) (Table 3).

**Table 3.** Description of the number of messages (*N* = 1941) sent by season, day and time of day.

Variables	*N*	%
Season		
Winter (January–March)	556	28.6%
Spring (April–June)	567	29.2%
Summer (July–September)	349	18.0%
Autumn (October–December)	469	24.2%
Day		
Sunday	279	14.4%
Monday	295	15.2%
Tuesday	281	14.5%
Wednesday	292	15.0%
Thursday	309	15.9%
Friday	252	13.0%
Saturday	233	12.0%
Time of the day		
Night	107	5.5%
Day	591	30.4%
Evening	1243	64.0%

### Content of the messages

Three main themes were formed to represent the content of the young people’s messages: ‘Social environment’, ‘Construction of self’ and ‘Health and wellbeing’. Quotes with IDs illustrate the content of each theme (Braun & Clarke, 2006).

### Social environment

Two subthemes represented social environment: 1) interpersonal relationships and 2) living environment.

*Interpersonal relationships* included descriptions of young people’s personal experiences with other people (e.g. parents, siblings, friends, peers, teachers, loved ones, romantic interests) or their observations of the relationships between other people (e.g. relationships between parents, friends or schoolmates).

“I had a quarrel with my best friend, who I had known for over 10 years. It felt pretty awful, and I was already thinking that I no longer have anything here on earth. … Well, luckily it didn’t take long before that quarrel between me and my friend was settled.” (ID 38899)

“Neither gave in and the situation developed into a quarrel that no one wanted. Mom and Dad had a fight, and it ended when Dad insulted mom with his words really badly. I know it because I was there.” (ID 38887)

Young people wrote about lacking in interpersonal relationships. They described loneliness and their hopes to have romantic relationships in the future. Some expressed that they did not want to have any interpersonal relationships. Some described the loss of an important person through separation or death, and the impact of that loss on other relationships. They also wrote about the violence that they had experienced, such as bullying, sexual harassment, rape or assault, and how it had influenced their life by making it difficult to trust or enter into new relationships.

“I miss a friend with whom I could immerse myself in deeper thoughts, or anyone with whom I could chat. Or maybe I just want to be important to someone.” (ID 3916)

“Due to unnecessary disputes and fights, in one month I’ve lost my best friend and a man for whom I had really strong emotions, as well as a few friends. And I have the feeling of being alone all the time. I don’t have anyone anymore.” (ID 39126)

“I’m also being bullied. Physically, it has usually been pushing, sexual harassment. Mentally, I have been bullied with words that are always burdening my mind… I no longer trust people or myself.” (ID 38885)

Young people also asked for advice on interpersonal relationships, like how to act or survive in them, and what to do in different relationships, for example, how to approach a romantic interest, how to survive conflict situations, or what to do with another person.

“Was I wrong? Didn’t she insult me? Didn’t she do the same to me, hurt and did not apologize? …What should I do? Should I swallow my pride and my need for independence, if not for my mother, for my own sake?” (ID 39734)

“How should I find out what he wants from me?” (ID 39731)

*Living environment.* Some messages contained descriptions of various places and circumstances of young people’s lives. These included information about home, school, work or vacation settings. Young people wrote about housing arrangements, where and with whom they lived, as well as how their housing was organised due to study, work or separation of parents. They also wrote about their hopes and fears of needing to move to another area, live with another parent or move away from home.

“I feel really bad at home. For example, last night as I was just falling asleep, my mom suddenly became enraged and came in, screaming at me… Do I have a chance to get into a foster family, if so, how can I get there?” (ID 38968)

“My parents are divorced: I live for a week with Mom and then a week with Dad… I have a really nice time with my brother when we are at our mom’s house every other week, but we cry while we are at our dad’s house.” (ID 39122)

The messages contained descriptions of the financial situations of the young people, and how that impacted their lives. They wrote about how their economic situation either enabled or hindered their independence to choose where to live or study. The young people asked for information regarding their housing and economic situations, such as their right to choose their place of residence and arrangement of living, or how and from where they could seek financial help.

“Thanks to my parents’ income, I won’t get any financial support until I turn 18. Mom and Dad have said that I can move out anytime I want, but I won’t get money from them.” (ID 39103)

“The problem is, during high school I want to move to my Dad’s, and that’s ok with him, but my Mom won’t let me move. My question is about how this matter goes legally, who has a say in this matter and who does not?” (ID 39129)

### Construction of self

Three subthemes represented the construction of the self: 1) past self, 2) present self and 3) future self.

*Past self.* The young people shared stories of past events that had shaped them. These stories included the young people’s childhood memories and what they remembered or had heard (e.g. their own illness or that of a parent). These writings included events that had happened years or months prior, such as a divorce between parents or diseases that had already been successfully treated. In these stories, the young people told about how they had accepted those situations and how going through them had affected the kind of people they had become.

“I’ve heard a lot of good things. That my mother has protected me from everything and made sure everything is fine. I’ve heard that my mother always watched my room when I was sleeping. And especially before my mother died, she had visited a lot just to watch me. I was really small when my mother died, but I think this has some meaning. I don’t know what it is, but maybe sometimes I’ll get to know it, or I won’t.” (ID 38937)

“For the first time in many months, I don’t feel any pain. Feeling tired but happy. I survived anyway. I wrote a previous text a year ago. I had been diagnosed with leukaemia and survival was not certain. Everything was overshadowed by huge fear of death, certainly due in large part to the death of my mother… I don’t have very many memories of my mother, but I remember being with my father a lot at home when my mother was in the hospital. … Because of this, I have a strong character and I don’t easily give up.” (ID 38959)

*Present self.* The young people shared their perceptions of themselves. They described themselves in terms of their personality, sexuality, capabilities, behaviour and self-worth. They expressed what kind of person they thought they were, for example, shy, loyal, or diligent. They perceived their likes or dislikes in a neutral way, such as hobbies, writing, music, and animals. Some wrote about their sexuality and what gender they represented.

“I’m actually a good and kind-hearted person, and therefore I was also surprised by my own actions.” (ID 39392)

“At the moment, I like to define myself as pansexual, but I am still a little unsure about it. Sometimes I wonder if I’ve just come up with the whole thing on my own. The idea of it feels good, and I could easily imagine myself in a romantic relationship with any sex—gender doesn’t matter.” (ID 38859)

The messages also contained perceptions of young people’s own capabilities, such as how they had or had not dared or been able to use their capabilities to talk to someone or refuse to do something. They shared their thoughts on what kind of picture of themselves they presented to others, and those kinds of thoughts affected their perception of themselves and their actions. Some described their own behaviour, such as how they had controlled or tried to control their emotions and actions. They told of their motivation or lack of motivation to do things like homework, schoolwork, or take care of themselves; or they described how they had tried to regulate different symptoms like anxiety, dejection, pain or sleeping problems in helpful (e.g. talking, writing, hobbies) or harmful (e.g. self-mutilation, self-hitting, eating control) ways. Young people also wrote about their self-worth in critical and positive ways, and about their experiences and attitudes towards their own body and thoughts.

“I recognize in myself that I’m a real daredevil, I want to try all kinds of stuff.” (ID 39138)

“I am happy to continue this because it’s great to get rid of the thoughts, etc. because I cannot talk at all about how I feel. If I try to talk about how it feels, etc., I can’t get a word out of my mouth.” (ID 39952)

“I have never loved myself. I really consider myself an ugly, fat and stupid person. The motivation for school also decreases and decreases, and there are a lot of absences, which in turn stresses me out terribly.” (ID 40569)

“I once again decided to write you a letter because I think it is the best way to vent feelings. Sometimes it’s safest to share your information only with people you don’t know personally.” (ID 39970)

“I had no idea how to handle my bad feelings, so I thought cutting myself would make it better. Well, it did.” (ID 39540)

“Feels like I do everything wrong, I guess I’m somehow bad or faulty. It feels that I’m an obstacle, annoying, useless and the beginning and end of all evil.” (ID 39571)

“Today, my self-esteem has improved a bit. I’m pretty happy with my body and my face with acne, which my mother fusses about.” (ID 38999)

*Future self.* The messages contained descriptions of young people’s perceptions of their future selves. Some young people expressed how they had confidence that in their future they would be able to get what they desired, such as health, independency, success in studies or parenthood. On the other hand, some conveyed that they could not see themselves in any role or even alive in the future, and that they did not have the ability to cope with the future.

“I love children so much and I’d like to be a mother and take care of them.” (ID 39780)

“I also have many dreams that I’d like to make come true. Some of them will take time, but I still want to make them work, like finding a nice job and getting a dog.” (ID 39091)

“I lost the bright flame for the future a long time ago. Or did it ever even light up? I want a new wind to take hold of me and carry me to a sturdy ground on which to build something. New dreams, new doors to open.” (ID39867)

### Health and well-being

Two subthemes represented health and well-being: 1) health-related problems and 2) experiences and perceptions of seeking and receiving help from professionals.

*Health-related problems.* Young people described their health problems as specific mental health illnesses and diagnosed or suspected physical sicknesses. Symptoms described included fatigue, pain, sleep problems, mood issues and mental health problems. Messages included descriptions about vehicle accidents, falls and sports injuries. They wrote about substance use, including tobacco and alcohol use. Writings about general health problems included the topics of unhealthy eating, lack of exercise, or disrepair.

“I’ve been diagnosed with depression, panic disorder and anxiety. … Medicines do not help; they have been tried. Instead, I’m an idiot, I smoke weed and tobacco and drink alcohol.” (ID 39157)

“I still get seizures, but much less often than when I was little… It wasn’t until a year ago that I got such a good medicine that I could even live a normal life in some way.” (ID 38939)

“We’d crashed into an oncoming car… I had a severe concussion and my back had broken.” (ID 38953)

“Lately I’ve been really tired and bit depressed.” (ID 40485)

“I’m about 27 kg overweight and it’s hard to lose weight.” (ID 39652)

The young people asked for information, help and advice related to health, adolescent development and sex, and what is normal or not normal, for example, regarding symptoms, height or weight, or ways to have sex.

“Is this kind of behaviour normal, or should I be worried since my mood is getting worse?” (ID 39337)

“Is it even possible that someone my age could have breast cancer?” (ID38794)

“What is sex? How do you have sex?” (ID 39374)

*Experiences and perceptions of seeking and receiving help from professionals.* Young people described from where and with whom they had sought help from the services available: for example, from health care services, social services, pupil and student welfare, or the third sector with a parent or school counsellor or by themselves. Young people also described if their experiences of seeking help had been easy or difficult, and how many people they must have met during the help-seeking process. They wrote about their experiences of receiving and providing help. Positive experiences included situations in which young people felt heard and understood by an empathic nurse or online counsellor. Negative experiences involved situations in which young people did not feel heard or understood, as well as experiences of breach of confidentiality resulting in a loss of confidence in professionals.

“I’m scared it’ll result in experiences similar to those I had with the school social worker and psychologist which, after all, did not help properly and my bad feeling and self-loathing continues.” (ID 39174)

“Anxiety began to come and self-mutilating started. The school counsellor referred me to the child guidance and family counselling centre, and now I am about to see a doctor.” (ID 38932)

“Thanks to that answer, I went to the school counsellor’s office. From there I was referred to a school psychologist, and now I now go there once a week. I can’t say if things are better yet. But now I know someone is here for me.” (ID 38918)

“Worst of all is that nobody listens to me or is willing to help.” (ID 39259)

Young people asked for information and advice about health-related confidentiality, for example, is it mandatory for professionals to tell parents about young people seeking help and using the services, or from where and how young people could get help with their mental health needs.

“Are the doctors obliged to confidentiality even with this kind of stuff [possibility of depression] even though I’m underage? And can the doctor inform that I’ve visited them or been tested?

And who or what should I even contact in this kind of situation?” (ID 39308)

### Factors associated with message patterns

Some statistically significant associations between background characteristics of young people, their messaging pattern and the content of the messages were found. Regarding the number of web messages sent, statistically significant differences were found between age and gender (Table 4). Those who were 15–17 years old sent more messages than those under 11 years old (*p* = 0.02, η^2^ = 0.01) or those who were 12–14 years old (*p* = 0.01, η^2^ = 0.02). A pairwise comparison further showed that males sent more messages than females (*p* = 0.03, η^2^ = 0.005). Regarding the length of the messaging period, a statistically significant difference was found in the residential area of the young people (Table 4). Those who lived in urban areas had longer messaging periods compared to those who lived in rural areas (*p* = 0.02, η^2^ = 0.02).

**Table 4.** The differences between background information and web messaging pattern.

Background variables	*N*	Mean	SD	Md	Mode	Effect size	*p* value^a^
Total number of messages^b^							
(*N* = 1354)							
Age						0.02^e^	<0.01
≤11	64	1.42	0.79	1	1		
12–14	440	1.67	2.40	1	1		
15–17	642	1.22	0.75	1	1		
≥18	208	1.60	2.80	1	1		
Gender						0.01^e^	0.02
Female	1178	1.39	1.76	1	1		
Male	119	1.61	1.45	1	1		
Other	57	1.96	3.57	1	1		
Residential						0.002^f^	0.1
Urban	957	1.35	1.38	1	1		
Rural	397	1.63	2.65	1	1		
Length of period of messaging^c^							
(*N* = 239)							
Age							0.02
≤11	18	27.17	47.50	5.5	5	0.02^e^	
12–14	105	53.52	72.69	18	5		
15–17	85	33.65	55.09	11	3^g^		
≥18	31	51.00	63.88	29	2^g^		
Gender						0.001^e^	0.96
Female	196	42.97	61.64	14	5		
Male	30	50.84	78.08	16	2^g^		
Other	13	76.94	76.94	11	1^g^		
Residential						0.02^f^	0.02
Urban	160	35.48	54.35	13	5		
Rural	79	61.7	78.81	20	5		
Number of words^d^ (*N* = 1941)							
Age						0.14^e^	<0.01
≤11	91	58.67	57.77	32	9		
12–14	736	176.99	174.25	126	54		
15–17	782	269.89	220.45	206	133^g^		
≥18	332	325.83	368.56	230.5	66d		
Gender						0.04^e^	<0.01
Female	1638	242.01	248.19	175	133		
Male	191	138.79	165.89	88	10^g^		
Other	112	284.92	238.94	211.5	8		
Residential						0.01^f^	<0.01
Urban	1288	240.64	219.76	177.5	9		
Rural	653	221.89	283.01	142	66^g^		

^a^ *p* values above 0.05 were not considered significant.

^b^ The number of messages sent by young people (*N* = 1354)

^c^ The length of period of messaging of young people (N = 239) who sent at least two messages.

^d^ The number of words used in messages (*N* = 1941).

^e^ Effect sizes (ES) estimated using epsilon^2^ (ε^2^).

^f^ eta squared (η^2^) with benchmarks <0.01small, <0.06 medium and <0.14 large effect.

^g^ Multiple modes exist, the smallest value is shown.

Associations were found between background information and the number of words used in each message (Table 4). First, young people in older age groups tended to send longer messages with more words than those in younger age groups. The difference between age groups was statistically significant in all pairwise comparisons (*p* = 0.01) except when comparing the 15–17-year-olds to those 18 years old and older (*p* = 0.72, η^2^ = 0.003). The effect size was large comparing the under 11 age group to all other age groups (η^2^ = 0.15–0.5). Second, the difference in message length was statistically significant: females (*p* = 0.03, η^2^ = 0.04) and those categorised as ‘other’ in terms of gender (*p* = 0.03, η^2^ = 0.3) wrote longer messages than males. Third, young people in urban areas sent longer messages than those who lived in rural areas (*p* = 0.01, η^2^ = 0.01).

Young people who sent at least two messages were typically 12–14 years old (104/239, 43.5%). Females (196/239, 82%) sent at least two messages more often than the other gender groups. Two-thirds of those who sent at least two messages lived in an urban area (160/239, 66.5%). A statistically significant difference was found between age and gender and whether young people sent one or at least two messages (Table 5). A pairwise test showed a statistically significant difference in the age group of 12–14-year-olds; they wrote at least two messages more often than 15–17-year-olds (*p* ≤ 0.001, OR 2.08) or ≥18 years old (*p* = 0.04, OR 1.81). No statistically significant differences were found between gender and how many messages were sent.

**Table 5.** Characteristics of participants who sent one (*N* = 1155) or at least two messages (*N* = 239).

	One message sent (*N* = 1115)		At least two messages sent (*N* = 239)			
Background variables	*n*	%	*n*	%	.(df) p^a^ Pearson chi	Cramer’s V
Age					25.724 (df 3), *p* ≤ 0.001	0.138
≤11	47	4.2%	17	7.1%		
12–14	336	30.1%	104	43.5%		
15–17	555	49.8%	87	36.4%		
≥18	177	15.9%	31	13.0%		
Gender					6.551 (df 2), *p* = 0.04	0.070
Female	982	88.1%	196	82.0%		
Male	89	8.0%	30	12.6%		
Other	44	3.9%	13	5.4%		
Residential					1.953 (df 1), *p* = 0.16	0.183
Urban	797	71.5%	160	66.5%		
Rural	317	28.4%	80	33.5%		

^a^ Chi-square test and *p* value.

### Factors associated with content

The young people’s messages mostly contained topics related to the main theme of ‘Social environment’ (1208/1354, 89.2%), especially about the subtheme of ‘Interpersonal relationships’ (1131/1354, 83.5%). The messages were most seldomly related to the main theme ‘Health and wellbeing’ (1008/1354, 74%) and the subtheme ‘Future self’ (423/1354, 31.2%) (Table 6). The fewest subthemes were addressed in messages sent by the youngest age group (Median 2) and males (Median 3) (Table 7).

**Table 6.** Description of the number of different main themes and subthemes that appeared in the young people’s (*N* = 1354) messages^a^.

Main themes	*N*	%
Subthemes		
Social environment	1208	89.2
Interpersonal relationships	1131	83.5
Living environment	861	63.6
Construction of self	1080	79.8
Past self	529	39.1
Present self	1007	74.4
Future self	423	31.2
Health and well-being	1002	74.0
Health-related problems	937	69.2
Experiences and perceptions of seeking and receiving help from professionals	568	41.9

^a^ One person could have written about more than one theme in one message.

**Table 7.** Description of background variables of young people (*n* = 1354) and number of the subthemes in their messages.

	Number of the subthemes						
Background variables							
	*N*	Min	Mean	SD	Max	Median	Mode
Age							
≤11	64	1	2.6	1.5	6	2	1
12–14	440	1	3.7	1.7	7	4	3
15–17	642	1	4.2	1.7	7	4	3
≥18	208	1	4.5	1.8	7	5	4^a^
Gender							
Female	1178	1	4.1	1.7	7	4	3
Male	119	1	3.3	1.8	7	3	2
Other	57	1	4.1	2.1	7	4	1
Residential							
Urban	957	1	3.9	1.7	7	4	3
Rural	397	1	4.1	1.8	7	4	5

^a^ Multiple modes exist. The smallest value is shown.

## Discussion

In this study, we aimed to describe web messaging patterns and the content of the web messages among young people using a national online service in Finland. In addition, factors associated with the messaging patterns and content were analysed. We found specific patterns in the use of web messaging and the content of the messages. Young people typically sent only one message, and most of messages were sent in the evening. The summer season was the period when the fewest messages were sent. Girls used messaging more actively than boys or those who identified as ‘other’ in terms of gender. The young people’s messages mostly contained topics related to the main theme of ‘Social environment’. The lowest number of different subthemes was found in the messages sent by the youngest age group and boys. In general, the use of web messaging varied among young people, as did the content of their messages. An analysis of the elements of web messaging offers novel insight, not only into the use of web messaging but also into young people’s own perspectives of their daily lives.

Typically, the young people in our study sent only one message to the national web messaging service, and a minority of the young people sent two message or more, some even up to 31 messages. Our results support previous findings where young people may just want to share, with a single message, their daily activities with someone, while a small group of young people is willing to share their complex life situation with professional in a long-term discussion.^5,6^ Previous studies have also found that young people with complex problems seek multiple counselling sessions.^48^ As long-term discussions can be sensitive, any delay in a counsellor’s response may cause a user to quit using the service.^49^ Therefore, it might be crucial in the future to identify young people who are in real need of mental health services, respond to their messages without delay and refer these young people to professional services.

In our data, messages were sent mostly in the spring, autumn and winter. These are typical periods of higher stress for young people because of school. This finding is in line with another study that showed that young people used a health information website the least in the summertime.^15^ In Finland, school summer holidays are between early June and mid-August, which might also have impacted web messaging. It is possible that summer holidays are less stressful for most young people. We also found that over half of the messages were sent during evening hours, after school. This finding is supported by previous studies showing that evening hours are the most common times for young people to use online services.^8,15,31^ In Finland, parents often work outside the home, and children come home to empty houses after their school days. Our results may indicate that young people need adults to share their thoughts with after school. If this opportunity is missing in a young person’s inner circle, they may seek contact with a stranger, someone who will listen and respond to them, even if delayed. In these cases, volunteer online messaging is a useful service to complement communication between young people and adults.

Previous studies regarding the users of web message online services show that boys have been the minority in online web services.^6–8,11^ This was also the case in our study. As in the previous studies, boys wrote shorter messages than girls.^8^ They also touched on fewer subthemes in their messages than girls and those who identified as ‘other’ in terms of gender. It has been discussed that boys might be less eager to write their worries in text format.^50^ They also seem to prefer to seek counselling over the telephone^7^ or face to face,^5^ although they have indicated that it is easier for them to use online services to get help with their mental health problems than to talk face to face.^18,19^ Therefore, there may be a need to develop an online service where young people can communicate via text, audio or video, like in Discord (see https://discord.com/).

The messages contained texts related to topics typical for young people on text-based online services, such as interpersonal relations and health-related problems.^6,8,11,15,16^ They often sought information, advice or help on these same topics. In addition, there were messages with stories, descriptions and thoughts about the young people themselves and their lives. In these messages, young people described and evaluated themselves by openly sharing their feelings and experiences about their self-confidence, self-worth, self-acceptance, competence, and ability.^44^ Previous studies have shown that it is easy for young people to present sensitive, emotionally or otherwise challenging topics in anonymous text-based online services.^7,11^ The ability of young people to, not only ask about but also to communicate about themselves and their lives through two-way web messaging should therefore be taken into account when developing health services for young people.^51^

### Limitations

Our study has a number of limitations that need to be taken into account. First, a service provider anonymised the messages, and the text data were transferred into the data format, which might have changed the original word count due to technical reasons. Second, the content of the messages was complex and varied widely. Therefore, we used thematic analysis, which considered not only individual words, for example, meanings, but also the context^25^ in which the words are used. We assumed that this would provide us with a comprehensive understanding of the issues raised in the messages. On the other hand, as the target group of the study was young people, we are not certain if the thematic analysis captured the depth of their situations. Third, we developed our data categorisation framework using thematic analysis and the codebook. However, we must question whether a sufficient number of messages was included in the development of the codebook. Based on the methodological literature, an analytical framework is complete when it works well, and no new codes are identified.^38,42^ In our study, this was the case after 30 messages. In addition, we evaluated a new set of 30 messages with ICR in a pilot test.^42^ Based on this process, we believe the qualitative analysis is reliable.

Fourth, it might be possible that interpersonal dynamics could have impacted coding negotiations.^36^ For example, a coder’s background, whether it be in education, working life or research, for example, might affect their way of thinking.^25^ In this study, the collaboration between researchers worked well; they were able to bring up any differing perspectives and find solutions through discussions, and they were used to working together. Fifth, the data were gathered on the basis of the use of multiple two-way messaging during the first year that the service was available, which might have included unique results that do not represent the current use of web messaging. On the other hand, study participants were similar in gender, age and residency compared to previous studies on text-based online services for young people.^5,8,10,11^

## Conclusion

This study offers information about web messaging among young people. The young people in our study mostly used web messaging once, and the content of messages varied from clear questions to complex descriptions and stories of the young people’s lives. Based on a variety of codes and categories in the messages, we can assume that web messaging offers a confidential communication channel through which young people can reflect and discuss their problems and difficulties with counsellors. However, further research is still needed to improve the knowledge about the usage and effectiveness of multiple two-way web messaging. The results of this study can be used to develop easily accessible online health services for young people.
